#  Trauma Registry of the Pan-American Trauma Society: One year of experience in two hospitals in southwest Colombia 

**Published:** 2016-09-30

**Authors:** Carlos A Ordoñez, Mónica Morales, Johanna Carolina Rojas-Mirquez, Francisco Javier Bonilla-Escobar, Marisol Badiel, Fernando Miñán Arana, Adolfo González, Luis Fernando Pino, Amadeus Uribe-Gómez, Mario Alain Herrera, Maria Isabel Gutiérrez-Martínez, Juan Carlos Puyana, Michael Abutanos, Rao R Ivatury

**Affiliations:** 1 Departamento de Cirugía, Facultad de Salud, Universidad del Valle, Cali, Colombia; 2 Hospital Universitario del Valle, Cali, Colombia; 3 Fundación Valle de Lili, Cali, Colombia; 4 Grupo de Investigación Epidemiología del Trauma y las Lesiones, Universidad del Valle, Cali, Colombia; 5 Instituto CISALVA, Universidad del Valle, Cali, Colombia; 6 Universidad ICESI, Cali, Colombia; 7 Department of Surgery, Division of Trauma, Critical Care and Emergency General Surgery, Virginia Commonwealth University Medical Center, Richmond, Virginia, USA

**Keywords:** Emergency care information systems, registries, data systems, trauma, wounds and injuries, trauma severity indices, occupational injuries, cause of death

## Abstract

**Background:**

Trauma information systems are needed to improve decision making and to identify potential areas of intervention.

**Objective:**

To describe the first year of experience with a trauma registry in two referral centers in southwest Colombia.

**Methods:**

The study was performed in two referral centers in Cali. Patients with traumatic injuries seen between January 1 and December 31, 2012, were included. The collected information included demographics, mechanism of trauma, injury severity score (ISS), and mortality. A descriptive analysis was carried out.

**Results:**

A total of 17,431 patients were registered, of which 67.8% were male with an average age of 30 (±20) years. Workplace injuries were the cause of emergency consultations in 28.2% of cases, and falls were the most common mechanism of trauma (37.3%). Patients with an ISS ≥15 were mostly found in the 18-35-year age range (6.4%). Most patients who suffered a gunshot wound presented an ISS ≥15. A total of 2.5% of all patients died, whereas the mortality rate was 54% among patients with an ISS ≥15 and a gunshot wound.

**Conclusion:**

Once the trauma registry was successfully implemented in two institutions in Cali, the primary causes of admission were identified as falls and workplace injuries. The most severely compromised patients were in the population range between 18 and 35 years of age. The highest mortality was caused by gunshot wounds.

## Abstract (es)

**Introducción:**

Los sistemas de información en trauma son requeridos para mejorar la toma de decisiones e identificar potenciales áreas de intervención.

**Objetivo:**

Describir el primer año de experiencia del registro de trauma en dos centros de referencia de trauma del suroccidente Colombiano.

**Métodos:**

Estudio realizado en dos centros de referencia de Cali. Se incluyeron pacientes con trauma o lesiones externas entre el 1-Ene y el 31-Dic-2012. Se recolectó información demográfica, relacionada con mecanismos de trauma, severidad (ISS) y mortalidad. Se presenta un análisis descriptivo.

**Resultados:**

Se registraron 17,431 pacientes. El 67.8% de los pacientes eran de género masculino con edad promedio de 30 (±20) años. Las lesiones laborales fueron causa de consulta a urgencias en un 28.2%, y las caídas el mecanismo de trauma más frecuente (37.3%). Los pacientes con ISS ≥15 en su mayoría se encontraban en el rango de edad de 18-35 años (6.4%). El 28% de los pacientes que sufrieron lesión por arma de fuego presentaron un ISS ≥15. El 2.5% de los pacientes murieron y aquellos pacientes con ISS ≥15 y lesión por arma de fuego presentaron mortalidad del 54%.

**Conclusión:**

una vez se logró implementar el registro de trauma en dos instituciones en Cali, se identificó que la principal causa de ingreso fue secundaria a caídas y las lesiones laborales. Los pacientes más severamente comprometidos están en el rango de población entre 18 a 35 años. La mayor mortalidad se presentó secundario a lesiones de causa externa por arma de fuego.

## Introduction

Trauma is a worldwide epidemic and is categorized as the primary cause of death in people younger than 45 years of age and the third highest cause of death overall. Trauma is responsible for approximately 1.6 million deaths per year 1, which is more than HIV, tuberculosis, and malaria combined 2.

In Colombia, trauma has been defined by violence, with a mortality rate of 5.8% from 2005-2010. Given the impacts of trauma and violence on the community and healthcare institutions, trauma has become a public health problem for the country, which has resulted in the implementation of preventive measures, such as "social communication to change behaviors" related to violence and trauma 3,4. 

Electronic information systems are more frequently used in high-income countries with the objective of creating an electronic registry of patients who suffer any type of trauma. Trauma registries are an important tool in health and policy areas because they allow the identification of all patient information (from personal data to the kinematics of the trauma, hospital outcomes, and discharge) and the development of strategies and prevention policies based on these data 2,5.

Similarly, electronic Trauma Registry systems have proven to be highly useful in improving the quality of care given to patients with externally inflicted wounds. These systems have allowed the analysis of data over the short and long terms, resulting in changes in protocols and care guidelines, estimation of costs, optimization of services, and the formulation of hypotheses that promote research in this area 6.

The first modern trauma registries date to the early 1970s in the United States. The first experiment was in Illinois, where a database now known as the National Trauma Data Bank of the United States was developed; this database is the keystone of the trauma system in that country 7.

Conversely, Latin America lacks reporting of emergency trauma visits, which are the most frequent events in the region 8. The most similar report concerns the trauma registry completed from 2002 to 2004 in San Fernando General Hospital of Trinidad and Tobago 9. In Colombia, strategies have been developed to monitor externally inflicted wounds from which the pertinent variables related to the history of these wounds can be cataloged 10.

Cali is the third most important city of the country, with more than two million citizens, and has been ranked as one of the most violent cities in Colombia. The homicide rate in Cali has fluctuated from 124/100,000 people in 1993 to 65/100,000 people in 2015 11,12. Strategies have been formulated as a result of this difficult social situation. One strategy is the restructuring of information systems to understand the dynamics of trauma in Cali and to begin to define the requirements of a trauma system for the city based on this information. To accomplish this goal, a meeting was held between the Pan-American Trauma Society (Sociedad Panamericana de Trauma, SPT) and Virginia Commonwealth University to implement the International Trauma System Development Program (ITSDP) in the city of Cali. This program is a virtual platform that allows actual data on trauma patient care to be obtained. This system was implemented in two trauma referral centers in the city to obtain quantitative data on the real situation, with the secondary objective of finding new alternatives for the care of patients with externally inflicted wounds and the creation of an Integrated System of Trauma Care. 

The objective of this study is to present a report of the first year of the trauma registry, to report the variables collected in two trauma referral centers in the southwest of Colombia, and to describe our experience with including one private center and one public center in a trauma registry system.

## Materials and Methods

This prospective study was performed in two healthcare centers in the city of Cali. After the SPT and Virginia Commonwealth University invited the health center to implement the ITSDP, a meeting was held amongst the institutions. The meeting confirmed the hospitals' commitment to having a minimum equipment infrastructure (each hospital had between 2 and 4 mini-computers and/or tablets and a wireless Internet connection for the input of information). In turn, SPT and ITSDP guaranteed 16 hours of training on the functioning of the electronic registry platform for the personnel who would be entering information. The meeting established that the data entry personnel should be from a technical level in the heath-care sector (defined as being a technician or student of pre-hospital care). In each institution, a coordinator with a professional medical profile and a statistical professional for the management of each institution's information were identified. Project financing for the first year was the direct responsibility of the healthcare institutions. Next, a pilot study was run between June and December of 2011 that was designed to identify problems in platform operation and to formulate strategies for data collection, which would ideally be real-time. Initially, attempts were made to gather data identifying all patients with any type of injury in real-time. Given the large volume of registrations, the entry criteria were defined as male and female subjects of all age groups who stayed for at least 6 hours of observation in either hospital. Defining the criteria consolidated the implementation of a registry that captured information on each admission in real-time, with the subject's information completed at the time of discharge from the hospital. Here, we present the information collected over a 12-month period from January 1, 2012, through December 31, 2012. The project was approved by the research ethics committees of each hospital and the Universidad del Valle.

The Trauma Registry contains information on sociodemographics, pre-hospital events, mechanism of injury, and the severity index. Patient severity was established using the injury severity score (ISS) to stage the injury severity into the following three categories: mild injuries with an ISS less than 9, moderate injuries with an ISS from 9-15, and severe injuries with an ISS equal to or greater than 15 13,14. We also recorded the ISS and TRISS ratings, hospitalization data, intraoperative information, clinical outcomes, discharge status, and mortality. This information is stored in a digital platform (available at https://www.pubapps.vcu.edu/itsdp-tr/) in which 244 variables can be found for each patient included in the registry.

### Sites

The hospitals involved were different in nature (one private and one public). Although Colombia has been working to develop electronic patient records in all healthcare centers, this system has advanced more in private than in public centers. The private university institution and the public university type institution are the primary referral centers on the local and departmental levels in southwest Colombia. These centers are characterized by their high level of complexity, resulting in recording the highest number of patients in emergency services.

The private institution reported on its electronic patient record system using the SAP software (SAP NetWeaver Business Client 1.0). The institution consists of 448 beds, with 120 in the intensive care units (ICUs), of which 10 are reserved for trauma patients. The center also has a unit for pediatric and adult recovery that serves approximately 10,000 trauma patients annually.

The public institution is a moderate to high complexity care center. Paper charts are used to record patient data in some services, and electronic patient records are in the process of implementation. This institution has 78 beds in its trauma unit, including 38 in the ICU, and an emergency department accounting for 195 out of 750 total beds in the institution. This hospital is referenced in southwest Colombia as part of the public network and serves 8,450 trauma patients annually. These two institutions care for over 50% of all injured patients in the city.

### Data analysis

Each of the hospital's statisticians performed an evaluation of the data quality. For the preliminary analysis, data that did not mention the status and discharge of patients were excluded. Univariate and bivariate descriptive analyses were performed by omitting the missing data. Categorical variables were described with absolute and relative frequencies, and continual variables were described with medians and percentiles (p25-p75). Information exported as a binary archive was processed with the statistical package STATA(tm) 13(r) (StataCorp, TX, USA).

The study was approved by the Ethics Committees (Institutional Review Board) of the Universidad del Valle and the participant institutions. 

## Results

The study registered 17,431 patients, with 40.6% (7,081) in the public institution and 59.4% (10,350) in the private institution. Of these patients, 67.8% were male. The average age was 30 (±20) years, with the highest percentage of patients (38.9%) between 18 and 35 years of age (6,788) and the lowest percentage of patients older than 80 years (2.6%; 448) (Table 1). 

**Table 1.** Sociodemographic data.

Demographics	HUV (n=7,081)	%	FVL (n=10,350)	%	General (n=17,431)	%
Male	5,244	74,1	6,568	63.5	11,812	67.8
Age in years (average ± SD)	30.1 (21.8)		29.8 (18.4)		29.9 (19.8)	
Age in years (ranges)						
<18	2,241	31.6	2,767	26.7	5,008	28.7
18 a 35	2,547	36.0	4,241	41.0	6,788	38.9
36 a 55	1,283	18.1	2,366	22.9	3,649	20.9
56 a 79	718	10.1	800	7.7	1,518	8.7
80+	272	3.8	176	1.7	448	2.6
No data	20	0.3	0	0	20	0.1
Glasgow on admission (ranges)						
<8	288	4.1	156	1.5	444	2.5
8 a 11	184	2.6	64	0.6	248	1.4
12+	6,432	90.8	10,112	97.7	16,544	94.9
No data	177	2.5	13	0.1	190	1.1
Place of event						
Streets and avenues	3,293	46.5	1,102	10.6	4,395	25.2
Dwelling	2,141	30.2	1,597	15.4	3,738	21.4
Place not specified	654	9.2	3,991	38.6	4,645	26.6
Other place specified	410	5.8	665	6.4	1,075	6.2
Sport and athletic areas	186	2.6	899	8.7	1,085	6.2
School, other public institutions or administrative areas	110	1.6	535	5.2	645	3.7
Industrial or construction areas	103	1.5	1,058	10.2	1,161	6.7
Service and commercial areas	82	1.2	351	3.4	433	2.5
No data	63	0.9	73	0.7	136	0.8
Farms	26	0.4	36	0.3	62	0.4
Residential institution	13	0.2	43	0.4	56	0.3
Social insurance	5,971	84.3	9,938	96.0	15,909	91.3
Social insurance plan						
Contributive	760	12.7	8,310	83.6	9,070	57.0
FOSYGA	583	9.8	1,213	12.2	1796	11.3
Not specified	95	1.6	43	0.4	138	0.9
Other	46	0.8	26	0.3	72	0.5
SOAT	609	10.2	263	2.6	872	5.5
Subsidized	3,878	64.9	83	0.8	3,961	24.9
Intentionality n (%)						
Physical abuse	11	0.2	4	0.0	15	0.1
Sexual abuse	5	0.1	2	0.0	7	0.0
Attempted suicide	39	0.6	34	0.3	73	0.4
Suspected violence	2,013	28.4	462	4.5	2,475	14.2
Work-related injury n (%)	394	5.6	4,519	43.7	4,913	28.2
Patients referred n (%)	5,196	73.4	605	5.8	5,801	33.3
Hospitalized subjects n (%)	3,979	56.2	1,229	11.9	5,208	29.9
Subjects requiring ICU n (%)	196	2.8	459	4.4	655	3.8
Suspected alcohol abuse n (%)	883	12.5	157	1.5	1,040	6.0
Mortality in emergency	114	1.6	34	0.3	148	0.8
Mortality overall	328	4.6	112	1.1	444	2.5

HUV: Hospital Universitario del Valle: Evaristo Garcia

FVL: Fundación Clinica Valle del Lili

Work-related injuries were the cause of the emergency consultation in 28.2% of the cases (4,913), representing 5.6% (394) of the cases in the public hospital and 43.7% (4,519) of the cases in the private hospital. Regarding the highest frequency locations of injury, 25.2% (4,395) of the injuries occurred in the streets and avenues and 21.4% (3,738) in the home. 

The most frequent mechanism of injury was falling, with 37.3% (6,794), followed in order of frequency by contusion/crushing at 11.6% (2,105), knife wounds at 11.6% (2,112), traffic collisions at 10.5% (1,906), and gunshot wounds at 8.2% (1,489).

Intentional injuries comprised 14.7% (2,570) of the total injuries, amongst which physical abuse, sexual abuse, attempted suicide, and suspected violence were featured. Of these intentional injuries, 29.2% (2,086) were registered in the public institution and 4.9% (502) in the private institution. 

Regarding the anatomical location of the injury, 28.1% (5,001) of the patients presented injuries of the upper limbs, followed by the lower limbs at 20.4% (3,634), multiple injuries (more than one anatomical location) at 18.2% (3,250), face at 10.5% (1,875), and head injuries at 8.2% (1,464).

A total of 88.1% (15,395) of the patients presented with an ISS <9, 7.1% (1,241) with a score of 9-15, and 4.5% (790) with a score ≥15. Regarding the relationship between the ISS and the age group, the majority of patients with an ISS ≥15 were between 18 and 35 years of age (6.4%; 433) (Table 2). Furthermore, 28% (417) of the gunshot wound patients presented with an ISS ≥15, whereas 92.2% (6.264) of the patients with fall injuries had an ISS <9 (Table 3).

**Table 2.** Severity of trauma according to age group.

Age/ ISS	<9 (n)	%	9-14 (n)	%	≥15 (n)	%	No data
<18	4,692	30.5	198	16.0	116	15.0	2
18 a 35	5,870	38.1	484	39.0	433	55.0	1
36 a 55	3,297	21.4	202	16.3	149	19.0	1
56 a 79	1,252	8.1	192	15.5	73	9.2	1
≥80	269	1.7	163	13.1	16	2.0	0
No data	15	0.1	2	0.2	3	0.4	0
Total	15,395		1,241		7901		5

ISS= injury severity score

**Table 3.** Severity of trauma according to type of injury.

Mechanism of trauma/ISS	< 9 (n)	%	9-14 (n)	%	> 15 (n)	%
Traffic injury	1,535	80.5	206	10.8	165	8.7
Gunshot wound	767	51.5	304	20.4	417	28.0
Knife wound	1,891	89.5	163	7.7	58	2.7
Burn	367	85.3	36	8.4	27	6.3
Poisoning	215	97.7	4	1.8	1	0.5
Fall	6,264	92.2	440	6.5	90	1.3
Contusion/Crushing/Suffocation	1,920	99.0	11	0.6	8	0.4
Assault	656	95.5	21	3.1	10	1.5
Other inanimate mechanical force	704	97.6	14	1.9	3	0.4
Traumatic contact with machines	457	95.0	22	4.6	2	0.4
Overexertion	347	98.9	4	1.1	0	0.0
Other, specified	68	81.9	7	8.4	8	9.6
Not specified	204	95.3	9	4.2	1	0.5

ISS= injury severity score

Regarding mortality, 0.8% (148) of the patients died while in emergency services. The overall mortality was 2.5% (444), of which 86.6% (381) of the cases were male. Additionally, the mortality rate was 54% (244/417) for patients with an ISS ≥15 and injury by gunshot, followed by 38.9% (35/90) for patients with an ISS ≥15 and a fall injury, 37.6% (62/165) for patients with an ISS ≥15 and a traffic-related injury, 27.6% (16/58) for patients with an ISS ≥15 and a knife wound, and finally 14.8% (4/27) for patients with an ISS ≥15 and burn injuries (Table 4).

**Table 4.** Specific mortality according to trauma severity and mechanism of injury.

Mechanism of trauma/ISS	< 9 (n)	%	9-14 (n)	%	>15 (n)	%
Traffic injury	1	0.1	5	2.4	62	37.6
Gunshot wound	11	1.4	18	5.9	225	54.0
Knife wound	0		3	1.8	16	27.6
Burn	1	0.3	2	5.6	4	14.8
Poisoning	1	0.5	0		1	100.0
Fall	13	0.2	21	4.8	35	38.9
Assault	1	0.2	1	4.8	6	60.0
Others	3	0.1	0		14	63.6

ISS= injury severity score

## Discussion

 In Cali, it was successfully implemented a tool that systematically collects information related to patients admitted with any type of injury to two referral hospitals in the region. Using this information system, an epidemiological profile of injuries was established in the city, which is a critical input for decision-making regarding the need of a Trauma System. The implementation of trauma registries have acquired recent importance around the globe, especially in low- and middle-income countries (LMIC) where a higher burden due to injuries exists. Close to 90% of injuries are occurring in LMIC, causing social and economic issues 6. 

Trauma registries not only provide information necessary for the development of new care protocols and clinical research but are also fundamental for the development of trauma systems, with a subsequent improvement in the quality of care 6. Therefore, the adequate and opportune implementation of trauma systems and registries can decrease mortality indices by improving care, especially in countries where trauma is a public health problem.

The statistical analysis of homicide violence from 2008 to 2011 in the city of Cali revealed alarming figures related to the city's security. Starting in 2007, there was an estimated annual increase of 5.4% in the number of homicides, which was equal to the annual increase of 6.9% in firearms as the mechanism of trauma 15. In the present study, the findings were that the wounds occurred primarily due to intentional infliction (14.7%) (Table 1). Regarding the mechanism of trauma, gunshot wounds represented a significant percentage of the mortality in this population and a higher injury severity index despite not being the most frequent mechanism of injury.

 Although the statistics, which for years have revealed the state of insecurity in Cali, and the evident need of implementing a trauma registry, the first pilot program of the first trauma registry in the city (International Trauma System Development Program-ITSD) did not begun until 2011 16. This program allowed the identification of the reasons for the consultation, mechanism of trauma, trauma index, mortality, and other variables. The first experiment in the application of the trauma registry in Cali city performed over a 3-month period showed that the majority of the patients were male and registered in the public institution, which continued to be the case in the present study. Likewise, in the three-month pilot test and in the experience of this study, a considerable number of patients with a high severity index evaluated using ISS were admitted due to gunshot wounds. 

In Cape Town, South Africa, Nicol *et al*. 17, in one year, showed that 4.8% (442/9,236) of the patients were admitted with gunshot wounds. This result contrast to the statistics in the present study, which showed approximately double the number of gunshot wounds found in the population, with 8.2% of patients (1,488/17,426) presenting with this mechanism of trauma.

In LMIC, the state of implementation of trauma registries is not different from Cali´s situation because the availability of these data collection systems in these countries is low. A review of the literature regarding experiences with trauma registries LMIC performed by O'Reilly *et al*. 18, showed that 76 of the 84 articles included in the review came from 47 trauma registries, mostly from Iran, China, Jamaica, South Africa, and Uganda. Furthermore, the study showed a large difference and lack of systematization regarding the collected variables. The majority of the studies collected data related to the demographics, event, wounds, care process, severity of the wound, and final results. However, some studies differed in the variables related to the injury severity index used and the disability index, which complicated comparisons between trauma monitoring systems 19 .

Other initiatives at the Latin American level have not progressed given the requirement for guaranteed resources to maintain the human equipment structures and technology over time. To date, this initiative has been able to continue due to efforts aimed toward acquiring resources via internal and external grants by the hospitals and universities that provide the programs in medicine and surgical specialties in both scenarios.

This one-year experience with the SPT trauma registry reached a total of 17,431 patients, including a significant percentage of patients between 18 and 35 years of age and mainly males. Similar to the aforementioned study by Nicol et al., a higher percentage of patients who were male and under 40 years of age were mostly affected by injuries caused by violence 17; this age group was most affected by this phenomenon in Cali 15. Therefore, it is necessary to emphasize prevention programs in this age group because they are the most vulnerable population to developing risk behaviors that contribute to traffic-related injuries and violence (both interpersonal and self-inflicted) 20.

### Limitations

One of the technical difficulties that affect the updating of the trauma registry database is the availability of sufficient personnel to collect the information. The ideal of real-time capture has proven difficult. The system resorted to retrospective collection, with consequent bias introduced by incomplete data and the availability of these data in the patient records. In one of the centers in which this study was performed, the digitalization of patient records was in progress, which at times complicated their readability with subsequent information loss.

Regarding the methodology, this study was an ambispective study that allowed the evaluation of behavioral tendencies in patients with trauma or external wounds in two trauma referral centers in the city of Cali. However, the results did not allow the identification of trends in the incidence or prevalence of an injury type and the most important clinical results, the evaluation of cause-and-effect relationships, and a comparative study using the data acquired from the two referral centers because the variables were not adjusted in the institutions. Knowledge of these trends is fundamental for the establishment of trauma-management criteria, referral and counter-referral systems, allocation of resources to hospitals in the public network, and implementation of a regional trauma system for the optimal and efficient management of injuries.

## Conclusions

Strategies to achieve the creation of an information system based on the implementation of a trauma registry in two referral centers in Cali provided results related to the achievement of maintenance over time and methods that could be used to generated more precise information from sites caring for the trauma patients. The main strategy is the collaborative alliance with international entities (in this case SPT/ITSDP) and the political will of the hospitals to maintain the registry.

 Regarding the epidemiological profiles of the injured individuals, the patients included in the trauma registry mainly belonged to the 18-35 age group, in which the implementation and tracking of quality-of-care improvement programs is imperative and should be focused on violence prevention and reducing risky behaviors. Additionally, although falls were the most common mechanism of traumatic injury, gunshot wounds did represent important rates of mortality and had a higher severity score.

The trauma registry in Cali is the first initiative that has been maintained over time and is positioned as the primary source of an information system at the level of the care network of a population.
